# Disease-Specific Vs Non-Specific Questionnaires on Septoplasty Outcomes

**DOI:** 10.22038/IJORL.2022.59117.3076

**Published:** 2022-05

**Authors:** Nuno Medeiros, Cristina Aguiar, Paulo Pina, Nuno Barros Lima, João Larangeiro, Artur Condé

**Affiliations:** 1 *Department of Otorhinolaryngology, Vila Nova de Gaia / Espinho Hospital Center, Portugal.*

**Keywords:** Nasal Septum [MESH], Surveys and Questionnaires [MESH], Quality of Life [MESH]

## Abstract

**Introduction:**

Validated questionnaires are a valuable tool in medical practice. The role of septoplasty in improving patients’ non-nasal symptoms and their quality of life is still controversial. The aim of this study was to determine the differences in outcome after septoplasty measured by a disease-specific questionnaire vs a general QoL questionnaire.

**Materials and Methods:**

A total of 50 patients underwent septoplasty and completed the SNOT-22 and the SF-36v2 questionnaires preoperatively and at 6 months post-op. Pre-op, post-op and variation for each domain in both questionnaires were calculated and compared with a measure of *self-reported health transition (Question 2 of SF-36v2) and* with the Minimal important difference (MID) for our sample.

**Results:**

SNOT-22 scores significantly improved for each specific question and for the total score. SF-36v2 showed a significant improvement in scores for mental domains (Mental Health, Role Emotional, and Vitality) but less so for the physical domains. MID for our sample was 4.2 points. Patients with variations greater than 4.2 in SNOT-22 total score (74%) revealed significantly better variations in *Physical Function, General Health, Social Function and Vitality.*

**Conclusions:**

Validated questionnaires are a fundamental tool for assessing outcomes of commonly performed surgeries in ENT. Disease-specific questionnaire showed improvement in scores for the majority of patients. The general QoL showed improvement only in Mental Domains. This may suggest that the impact of septoplasty in patient’s general health might not be significant.

## Introduction

Nasal septum deviation is one of the most frequent findings in the nasosinusal examination, present in around 19-65% of the patients (1,2). Septoplasty is considered its definitive treatment, and it is one of the commonest surgeries performed by ear/nose/throat (ENT) surgeons (3). However, as the severity of the deviation is not a good predictor of nasal obstruction, surgical outcomes are somewhat unpredictable with short-term results being better than long-term ones (4,5). 

Symptoms such as snoring, olfactory impairment or hyponasality may also be present, which may impact the patient’s quality of life (QoL). Despite this, changes in general QoL after septal surgery are still uncertain(6). Even with objective or patient-reported improvement in nasosinusal complaints(7), the short and long-term benefits in the general physical and mental health of the patient have yielded conflicting results in most studies.

The standardized recollection of surgical outcomes using validated questionnaires is a valuable tool in current medical practice. These allow the evaluation of the benefit of surgical procedures; the improvement in selecting patients; the evaluation of predictive factors for better surgical outcomes. 

In a rhinology consultation, both disease-specific and general quality of life questionnaires are used. SNOT-22 is a questionnaire created for the assessment of the impact of surgery in the management of chronic rhinosinusitis (8), as well as in the evaluation of other chronic nasal pathologies. Buckland et al (9) used this instrument to assess septoplasty outcomes and found that 71% of patients reported a post-surgical score improvement. SF-36v2(10) (Quality Metric) is a questionnaire of general QoL that encompasses eight different domains in health which can be divided, as per component analyses, in two dimensions: a physical and a mental dimension. These two are distinct, and an overall score should not be obtained, according to the creators of the questionnaire (11). 

The main objective of the present study is to ascertain the correlation between SNOT-22 score and general-health domains after septoplasty. The study then aims to understand which nasal symptoms were modified by surgery, and also which non-nasal domains had significant variation.

## Materials and Methods

This was a prospective cohort study including all patients who were admitted for septoplasty in Centro Hospitalar Vila Nova de Gaia/Espinho. The study adopted the following inclusion criteria: 1) adults aged 18 years or above; 2) who underwent a ENT-consultant-led surgery; 3) who fully completed both questionnaires, in two moments: pre-surgically and at 6 months post-surgery. Exclusion criteria included: 1) chronic rhinosinusitis with/without nasal polyposis; 2) patients with history of previous nasal surgery; 3) if requiring simultaneous rhinoplasty/uvulopalatoplasty and 4) those with background of septal perforation.

All patients admitted for septoplasty between September 2018 and June 2019 were invited to participate in this study. Written informed consent was obtained and ensured throughout the study, which was approved by the Hospital Ethics Committee (Protocol 318/2019) prior to data collection. All participants self-completed the Portuguese validated version of both questionnaires (12,13), both pre-surgically and at 6 months post-surgically. In the Sino Nasal Outcome Test 22 (SNOT-22), patients are asked to rate 22 different symptoms related to both nasal and general health on a score from 0 to 5, with a higher score representing higher impairment. In the SF-36v2, eight different domains are studied: physical functioning (PF), role physical (RP), bodily pain (BP), general health (GH), vitality (VT), social functioning (SF), role emotional (RE), and mental health (MH), with different scoring in each question, originating a score from 0 to 100. 

The adopted surgical technique was standardized and included a hemitransfixion incision followed by an elevation of the septal mucoperichondrium in both sides, addressing all areas of deviation and reshaping or removing the deviated part of the cartilage. All patients also underwent inferior turbinoplasty to reduce their size. The anterior nasal packing placed at the end of the operation was removed after 48 h, when applicable. None of the patients suffered major complications. All operations were performed by ENT consultants (to reduce surgery skill as a possible bias), who were blinded to the patients’ scores both before and after the treatment and did not participate in neither the data collection nor the data analysis. 

Score variation for each item of both scores was analyzed by the Stuart-Maxwell test, while total scores were evaluated by paired student t-test. A paired student t-test was also used for analyzing the score variation of each domain of SF36v2. The total score was not calculated as it has no clinical value. An independent variable student t-test was therefore used to compare both questionnaires. The ‘minimally important difference’ (MID) defines the difference in a score that is clinically significant. SF-36v2 contains a patient-reported transition rating scale comparing pre and postoperative health (1=‘much better’, 2= ‘slightly better’, 3 = ‘same’, 4 = ‘slightly worse’, 5= ‘much worse’). The mean change in SNOT22 scores for patients in each of the transition rating categories was calculated. MID was defined as the change in score for those who reported their symptoms to be ‘slightly better’ minus the mean change score for those who reported their symptoms were ‘same’.

 IBM SPSS Statistics 26.0® for Macintosh® was used for statistical analysis, with a fixed value of p<0.05 for statistical significance.

## Results

A total of 57 patients were selected to take part in the study, with 50 patients being included in the final analysis after filling both questionnaires in both consultations. 

17 patients (34%) were females, with a mean age of 40 +/- 14.15 years old (min =18; max= 70 years old) (Table 1). No post-surgical complications were reported.

**Table 1:** Clinical and demographic characteristics of the study population (n=50)

	Mean	SD
**Age**	40.0	14.15
	*n*	*(%)*
**Sex** Male Female	33 17	(66.0%) (34.0%)
**Smoking** Yes No	9 41	(18.0%) (82.0%)
**OSAS** Yes No	5 45	(10.0%) (90.0%)
**Asthma** Yes No	7 43	(14.0%) (86.0%)

Table 2 reports the pre-surgical mean, post-surgical mean, and variation for each individual question and for the total score of SNOT-22, divided by gender. Some questions showed pre- and post-surgically gender differences. However, no differences in variation in scores were noted between questions. Every question suffered a statistically significant variation in its score, with the greatest variation found in question 22 (blockage/congestion of nose). Linear regression models for each question variation, showed no correlation with age. Patients reported improvement in nasal obstruction in 86% of cases. Non-nasal symptoms, in general, only improved in 52% of patients. Table 3 describes the pre-surgical means, post-surgical means, and variation for each domain of SF-36v2. Some groups showed pre- and post-surgically gender differences. However, no differences in score variation were noted between groups.Only Mental Health, Role Emotional, and Vitality reached significant variation, all of them being domains of the Mental dimension of this questionnaire. Linear regression models for each question variation, showed no correlation with age.

**Table 2:** Pre-operatory, post-operatory, and variation mean values for each SNOT-22 item, sorted by gender

	**Pre-op mean**					**Post-op mean**						**Variation**						**p-value**
	**Women**		**Men**			**Women**		**Men**			**Women**			**Men**				
**Q** **uestions**	**M**	**SD**	**M**	**SD**	**p**	**M**	**SD**	**M**	**SD**	**p**	**M**		**SD**	**M**	**SD**	**p**		
1.Need to blow nose	2.53	1.59	2.33	1.29	0.64	1.65	1.54	1.42	1.17	0.57	0.88		1.73	0.91	1.40	0.95		<0.001^*^
2.Sneezing	3.06	1.35	2.24	1.23	0.04^*^	1.71	1.45	1.33	1.36	0.37	1.35		1.87	0.91	1.28	0.39		<0.001^*^
3.Runny Nose	2.65	1.62	2.03	1.57	0.20	1.47	1.33	0.97	1.08	0.16	0.71		0.47	0.79	0.42	0.53		<0.001^*^
4.Cough	2.41	1.54	1.42	1.20	0.02^*^	1.00	1.32	0.76	0.94	0.46	1.41		1.97	0.67	1.24	0.17		<0.001^*^
5.Post nasal discharge	2.59	1.00	1.94	1.50	0.12	1.12	1.41	1.27	1.44	0.72	1.47		1.50	0.67	1.41	0.07		<0.001^*^
6.Thick nasal discharge	2.18	1.63	1.73	1.66	0.37	1.00	1.41	0.73	0.94	0.42	1.18		1.88	1.00	1.50	0.72		<0.001^*^
7.Ear fulness	3.06	1.25	1.88	1.50	0.01^*^	2.00	1.37	1.12	1.19	0.02^*^	1.06		1.92	0.76	1.50	0.54		0.001^*^
8.Dizziness	1.94	1.78	1.15	1.46	0.10	0.82	1.13	0.52	0.94	0.31	1.12		1.76	0.64	1.22	0.32		<0.001^*^
9.Ear pain/pressure	2.24	1.48	0.97	1.36	0.004^*^	1.12	1.17	0.30	0.64	0.002^*^	1.12		1.76	0.64	1.22	0.33		0.001^*^
10.Facial pain/pressure	2.47	1.66	1.15	1.35	0.004^*^	0.88	1.32	0.52	0.97	0.27	1.59		1.94	0.64	1.14	0.08		<0.001^*^
11.Difficulty falling asleep	2.47	1.77	1.82	1.70	0.21	1.18	1.33	0.91	1.38	0.51	1.29		1.53	0.88	1.47	0.36		<0.001^*^
12.Waking up at night	2.88	1.69	2.06	1.71	0.11	1.82	1.29	1.21	1.45	0.15	1.06		1.56	0.76	1.41	0.49		<0.001^*^
13.Lack of a good night’s sleep	3.12	1.65	2.55	1.62	0.25	1.94	1.52	1.27	1.59	0.16	1.18		2.16	1.15	1.89	0.97		<0.001^*^
14.Waking up tired	3.41	1.42	2.15	1.37	0.004^*^	2.29	1.49	1.48	1.52	0.08	1.12		1.58	0.67	1.71	0.37		0.002^*^
15.Fatigue during the day	3.29	1.36	1.97	1.38	0.002^*^	2.24	1.48	1.39	1.17	0.03^*^	1.06		1.71	0.58	1.71	0.35		0.005^*^
16.Reduced productivity	2.65	1.46	1.48	1.25	0.005^*^	1.29	1.26	0.91	1.07	0.26	1.35		1.54	0.58	1.71	0.10		0.001^*^
17.Reduced concentration	2.65	1.54	1.72	1.46	0.04^*^	1.41	1.12	0.91	1.10	0.14	1.24		1.99	0.82	1.42	0.40		<0.001^*^
18.Frustrated/restless/irritated	3.00	1.58	1.58	1.44	0.002^*^	1.53	1.38	1.06	1.00	0.17	1.47		1.91	0.52	1.42	0.05		0.001^*^
19.Sad	2.24	1.56	1.03	1.29	0.005^*^	1.35	1.37	0.45	0.71	0.02^*^	0.88		1.87	0.58	1.39	0.51		0.004^*^
20.Embarassed	1.71	1.83	0.55	1.23	0.01^*^	0.65	1.12	0.18	0.39	0.11	1.06		1.78	0.36	1.32	0.17		0.009^*^
21.Sense of taste/smell	2.06	1.48	2.12	1.82	0.51	0.59	0.87	1.09	1.28	0.15	1.47		1.55	1.03	1.24	0.32		<0.001^*^
22.Blockage/congestion of nose	3.47	1.42	3.24	1.32	0.58	1.59	1.37	1.21	1.29	0.35	1.88		1.41	2.03	1.42	0.73		<0.001^*^
Total	58.06	24.40	39.12	19.08	0.004^*^	30.65	19.50	21.28	13.85	0.06	27.41		30.21	18.15	19.22	0.19		<0.001^*^

Independent t-test between genders in each section, and Stuart-Marxell test for total variation. M = mean; SD = standard deviation;p = P-value * = P<0.05

**Table 3:** Pre-operatory, post-operatory, and variation mean values for each SF-36v2 domain, sorted by gender

	**Pre-op mean**					**Post-op mean**					**Variation**					**p-value**
	**Women**		**Men**			**Women**		**Men**			**Women**		**Men**			
**Q** **uestions**	**M**	**SD**	**M**	**SD**	**p**	**M**	**SD**	**M**	**SD**	**p**	**M**	**SD**	**M**	**SD**	**p**	
1. Physical function	68.53	22.41	85.76	14.58	0.002^*^	80.00	17.23	86.79	19.64	0.23	11.48	23.70	1.03	20.70	0.11	0.15
2. Role physical	46.18	22.54	65.22	16.71	0.001^*^	54.12	25.99	65.28	19.58	0.13	7.94	33.73	-0.84	21.97	0.34	0.57
3. Bodily pain	67.35	25.18	74.55	20.90	0.29	66.03	27.68	80.65	21.54	0.05	-1.32	35.58	6.11	25.29	0.40	0.38
4. General Health	73.90	19.04	76.70	19.28	0.63	79.04	26.23	81.40	25.47	0.76	5.15	22.99	4.70	25.82	0.95	0.17
5. Mental Health	50.00	18.11	67.58	16.45	0.002^*^	67.94	23.79	75.44	17.32	0.10	17.94	20.62	7.85	22.11	0.13	0.001^*^
6. Role Emotional	56.86	28.14	81.57	22.61	0.21	72.55	32.51	87.14	20.02	0.11	15.59	38.85	5.58	27.93	0.30	0.048^*^
7. Social Function	64.71	23.06	81.82	15.34	0.03^*^	77.94	22.33	80.58	21.12	0.68	13.24	23.58	-1.24	23.46	0.05	0.28
8. Vitality	46.32	12.90	56.44	18.32	0.05	55.15	18.39	64.67	18.92	0.10	8.82	19.52	8.23	21.66	0.93	0.007^*^

Independent t-test between genders in each section, and paired t-test for variation. M = mean; SD = standard deviation; p = P-value * = P<0.05

Question 2 of SF-36v2 was compared with SNOT-22 score variation in Table 4. “Much better” has a statistically significant higher score than the other options. There is no significant difference between variation scores for “slightly better”, “same” or “slightly worse”. With these results, the MID of our sample was calculated at 4.2 points.

**Table 4:** Mean values of SNOT-22 variation, divided into 5 groups according to the answer to Question 2 of SF-36, i.e., self-reported health transition. n = number of patients; M = mean; SD = standard deviation

	**SNOT-22 variation**		
**Question 2**	n	M	SD
Much better	15	33,54	7,44
Slightly better	21	18,38	4,12
Same	11	14,18	6,45
Slightly worse	2	13,50	8,50
Much worse	1	-7,00	0,00

**Fig 1:**
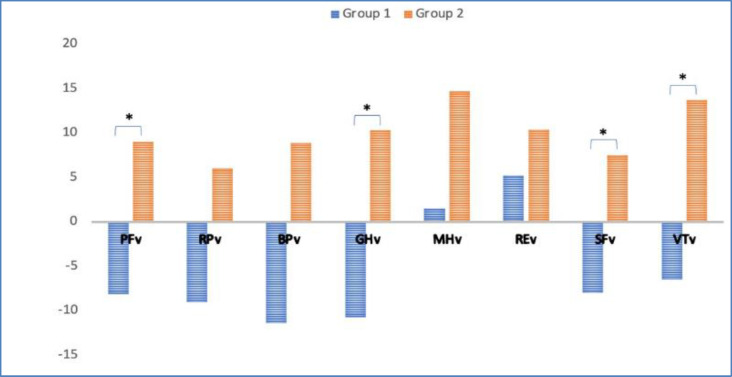
Pre- and 6-month postoperative SF-36 scores for each domain, divided into two groups according to the mean change in SNOT-22 values. Group 1(blue): change in SNOT-22 is <4.2 points. Group 2(orange): change in SNOT-22 is ≥4.2 points. PFv – Physical Function variation; RPv – Role Physical variation; BPv – Bodily Pain variation; GHv – General Health variation; MHv – Mental Health variation; REv – Role Emotional variation; SFv – Social Function variation; VTv – Vitality variaton; * P<0.05

Figure 1 compares SF36v2 variation for each domain between two groups: SNOT-22 variation of <4.2 points and >4.2 points, respectively. In this figure, it is notorious that both groups have very different SF-36v2 score variations, with four domains achieving statistical significance: Physical Function, Bodily Pain, Vitality, and Social Function.

## Discussion

The current study has shown that disease-specific symptoms might improve with septoplasty. General QoL, however, did not improve in all the domains measured in SF-36v2, despite demonstrating a significant improvement in most mental dimension domains. Disease-specific questionnaires such as SNOT-22, nasal obstruction septoplasty effectiveness study (NOSE) (4), or Rhinosinusitis Quality of Life Survey (RhinoQOL) (14) are widely used in ENT practice. In our sample, disease-specific QoL significantly improved in the post-operative period using the SNOT-22 questionnaire. This questionnaire was first introduced to evaluate surgical outcomes in chronic rhinosinusitis, but it is widely used for other rhinologic surgeries, including septoplasty (9,15). 

Other studies also reported significant improvement with septoplasty using SNOT-22 (9,16). In comparison, our sample had a higher pre-surgical mean score (45.6 points). In terms of post-surgical variation, Buckland et al (9) reported a mean variation of only 4 points in their sample. Our sample showed higher variations with surgery (21.3 points). Nasal obstruction improved in 86% of our patients at 6-months postoperatively. This is a higher percentage than what is reported by most studies, where improvement ranges between 70-80% (6,9,17,18). Non-nasal symptoms, also assessed by this questionnaire, improved in a smaller percentage of patients (52%). Similarly, Pannu et al. found non-nasal symptoms to improve only in 49% of cases at 4 months post-op (17). If we consider the minimally important difference classically stated by Hopkins et al(8) as 8.9 points, 62% of our patients achieved this variation. Hopkins et al.’s study comprised answers from 2043 patients submitted to nasosinusal surgery, however sole septal surgery patients were excluded from their analysis. A similar study with 52 patients was conducted in the Portuguese population with a much higher MID of 28 points (12). To date, no studies had been conducted in the Portuguese population assessing the MID in septal surgery. In the current study, the minimally important difference was 4.2 points, with 74% of patients reaching this difference. 

The correction of nasal symptoms might impact patients’ mental and physical status. SF-36v2 is a general health status questionnaire that, albeit not specific for septoplasty outcome evaluation, can offer relevant insight into general QoL changes (10). 

According to this study, three of the four parameters included in the mental dimension (mental health, role emotional, and vitality) showed significant improvement in the post-operative period. However, none of the physical dimension parameters achieved statistical significance. This suggests that septoplasty may have a positive effect in the mental health domain, although a benefit in physical health is harder to attain. In similar studies, results are conflicting when analyzing general QoL: the retrospective study by Simsek et al. reported no significant difference in all domains; whereas a recent prospective study by Erdivanli et al (7), showed improvement in all domains except for Bodily Pain and Social Function. 

Other tools for determining general QoL were also used in different studies: Valsamidis et al (19) showed no improvement in general QoL with septoplasty using the Glasgow Benefit Index; Naraghi et al (20) found a profound effect on the general QoL with this surgery, using a Modified Health-Related Quality of Life tool; and Hytonen et al (16), in turn, using the 15D tool, reported worsening of general QoL with surgery.

SF-36v2’s second question measures *self-reported health transition. When comparing these answers to SNOT-22 scores, as stated in *Table 3*, patients who replied “much better” have significatively higher score variation than patients who replied a slight improvement, no improvement or worsening. *

*However, score variation between the groups “slightly better”, “same” or “slightly worse” were not significantly different. This fact demonstrates that similar score variations with surgery might account for different perceptions of change in health status, highlighting the unpredictability of such intervention.*

*When comparing the results for SF-36v2 with the calculated MID, patients can be divided into two groups, as stated in* Figure 1 *.*

*Thus, there were significant differences in the variation found in the domains of Physical Function, General Health, Social Function, and Vitality. As such, the group that achieved a clinically relevant SNOT-22 variation, had it more prominently in these domains when compared to the other group. Hytonen et al (* 16 *) performed a similar comparison between SNOT-22 variation and 15D scores, using the limit of 8.9 points as stated by Hopkins et al. They found a significant change on 15D score in the >8.9 group of patients, with no 15D score change in patients with <8.9 points of variation.*

*The current study presented some methodological shortcomings. For example, it included patients recruited in one center and only measured short-term outcomes (6-months post-operatively).*

*It is well known that benefits from septal surgery might decrease after 6 months post-op. Despite the fact that every eligible patient was invited to participate, 12.8% were lost for follow-up.*

*These patients might have had more or less severe disease than the analyzed group, which limits the external validity of our results. *Moreover, gender, age and surgical skill are three of the most important possible confounders in our analysis. After gender stratification, and albeit some noted differences in pre- and post-surgical scores, variation was not influenced by this variable. 

A correlation was also not found between age and score variation in both questionnaires. However, surgical skill was controlled in our sample by only including patients who were operated by ENT consultants with more than 5 years of experience.

*Long-term and multicentric studies might be beneficial for assessing the real effect of septal surgery in general health, and also in determining population-based cutoffs for the most used questionnaires, which may improve patient selection.*

## Conclusion

When comparing scored obtained by disease-specific questionnaires at pre-operative and 6-months post-operative periods, the current study showed score improvement for the majority of patients. 

The results obtained for General QoL questionnaires emphasized an increase in scores relating to mental health domains but less so to the physical domains. Septoplasty outcomes may be sometimes unpredictable, with similar SNOT-22 variations being present in patients with different perceptions of health transition at 6 months post-op. This study also corroborates the importance of assessing outcomes using validated tools.
